# Counselling about the Risk of Preterm Delivery: A Systematic Review

**DOI:** 10.1155/2017/7320583

**Published:** 2017-08-07

**Authors:** Laura Pedrini, Federico Prefumo, Tiziana Frusca, Alberto Ghilardi

**Affiliations:** ^1^Department of Clinical and Experimental Sciences, University of Brescia, Brescia, Italy; ^2^Department of Obstetrics and Gynaecology, ASST Spedali Civili di Brescia, Brescia, Italy; ^3^Department of Obstetrics and Gynaecology, University of Parma, Parma, Italy

## Abstract

We aimed to describe the outcomes of counselling for preterm delivery. PubMed, Embase, and PsycInfo were systematically searched (from 2000 to 2016) using the following terms: counselling, pregnancy complications, high-risk pregnancy, fetal diseases, and prenatal care. A total of nine quantitative studies were identified, five randomized and four nonrandomized. All studies were conducted in the USA, and half of them were based on a simulated counselling session. Two main clinical implications can be drawn from the available studies: firstly, providing written information* before* or* during* the consultation seems to have a positive effect, while no effect was detected when written material was provided after the consultation. Secondly, parents' choices about treatment seemed to be influenced by spiritual-related aspects and/or preexisting preferences, rather than by the level of detail or by the order with which information was provided. Therefore, the exploration of parents' beliefs is crucial to reduce the risks of misconception and to guarantee choice in line with personal values. More research is necessary to validate these findings in cross-cultural contexts and in real world settings of care. Moreover, the centeredness of conversations and the characteristics of the clinician involved in counselling should be addressed in future studies.

## 1. Introduction

Pregnancy complications that predispose to preterm delivery represent a traumatic event for parents, and the quality of information provided to them during the antenatal period is crucial [1]. Healthcare professionals involved in counselling of mothers at risk of preterm delivery often report emotional demand and ethical dilemmas [2–4]. Indeed, preterm birth is associated with neonatal mortality, morbidity, and poor neurodevelopment, especially for lower gestational ages. Moreover, the unpredictable course and/or the sudden burden of the conditions predisposing to preterm delivery make the counselling even more difficult [5].

Clinicians are encouraged to provide complete information to parents and to engage them in shared decision-making by discussing their values and expectations [6]. Some issues support the emphasis on communication: firstly, the paternalistic model of patient-physician relationship has been progressively abandoned during the last decades [7]; secondly, mothers of premature infants are not always aware of the potential long-term problems [8]; finally, constructive communication and positive relationship with the medical staff are of the main determinants of satisfaction expressed by women with high-risk pregnancies [9, 10].

Shared decision-making (SDM) had been advocated as the optimal communication strategy for patient-centered care, particularly when there are important decisions to be made, and treatment options exist with different outcomes and substantial uncertainty [7, 11]. Patient-centered communication is based on a symmetric and collaborative relationship between patient and healthcare professionals [7]. According to this model, specific interpersonal skills and distinct steps during the consultation have been identified and they were shown to be effective strategies for the establishment of a trustful relation between patient and healthcare professionals [7]. The essential elements of a patient-centered consultation have been outlined [12], and educational intervention was shown to be effective in improving the communication skills of healthcare professionals [13]. Despite these efforts, clinicians often express concerns when the patient-centered model has to be translated into daily clinical practice [14, 15].

Previous studies showed that counselling approaches for preterm delivery are neither univocal nor explicitly defined, particularly regarding some of the contents of discussion (i.e., long-term outcomes, social and ethical issues) [16–18]. In addition, the overall setting of counselling (i.e., the place, the number of sessions, the tools, and the professionals to be involved) constitutes another relevant issue deserving attention. In a sample of mothers who received a diagnosis of fetal malformation, it was found that the level of maternal anxiety after delivery was inversely correlated to the number of prenatal consultations [19]. Moreover, as concerns the technical aspects of counselling, it should be considered that professional of different disciplines showed significant discrepancies in the knowledge about prematurity, as well as in the attitudes about the management of a pregnancy at risk of preterm delivery [20, 21]. Moreover, nationwide studies showed heterogeneous practices about the professionals involved in counselling [16, 17].

Considering the aforementioned difficulties as well as the heterogeneity in counselling practices, an evidence-based approach is useful for the development of effective counselling. This could be helpful for medical staff in planning the procedure of counselling for preterm delivery risk, allowing answering the following questions: Is the patient-centered model adopted during counselling for preterm delivery risk? Is there evidences about the efficacy of the patient-centered model during counselling for preterm delivery risk?.

The aim of this study was to systematically review quantitative studies on counselling for preterm delivery, in order to (1) assess the quality of the studies, (2) describe the methods of counselling reported, with particular regard to their adherence to the patient-centered model, and (3) assess the outcomes of the practices of counselling (both for parents and for professionals).

## 2. Materials and Methods

### 2.1. Review Protocol

The systematic review was conducted according to a prospective protocol and in accordance with the Preferred Reporting Items for Systematic Reviews and Meta-Analyses (PRISMA) statement [22]. The study was registered with the Prospective Registering of Systematic Reviews (PROSPERO) database (Registration number: CRD42014007123).

A systematic search was performed in MEDLINE, Embase, and PsycInfo, using combinations of the relevant medical subject heading (MeSH) terms, key words, and word variants for “Counselling”, “Pregnancy Complications”, “High-Risk Pregnancy”, “Fetal Diseases”, “Prenatal Care”, and “Prenatal Diagnosis”. Detailed search strategies are reported in Table 1. It had to be noted that “Preterm delivery” was not mentioned because according to the MeSH classification it is included under “Pregnancy Complications”. The search included articles that were published from January 2000 to December 2016, because the conference leading to the acknowledgment of the patient-centered model (i.e., Kalamazoo Consensus Statement) took place in May 1999 [12]. Reference lists of the articles identified using the search were scrutinized to further identify relevant articles.

### 2.2. Inclusion Criteria

Two authors independently analyzed the records of the searches. Studies were included if they satisfied the following criteria:

1. Being in English language
2. Having original data
3. Focusing on communication to women/parents at risk of preterm delivery.

Discordance between the two authors was resolved by consensus.

### 2.3. Data Extraction

A standardized data extraction form was completed for each included study, reporting aim of the study, study design, setting, year of publication, participants, procedure of counselling (i.e., professionals involved, use of tools, contents of discussion, timing and sessions, use of specific tools, and communication style), outcome, and main study results.

Data were independently abstracted by two authors, and any discordance was resolved by consensus. As we expected the included studies to be diverse in terms of design, setting, interventions, and outcome measures, a narrative synthesis was planned [23].

Adherence to the patient-centered model was determined based on the following criteria: (i) the authors state that counselling was planned according to the patient-centered model; (ii) the Kalamazoo Consensus Statement [7] is explicitly cited when the counselling procedure is described and/or the centeredness of counselling is measured by specific tools developed to assess the centeredness of the consultation.

### 2.4. Methodological Quality Assessment

The quality of study was assessed by two authors using the Cochrane quality assessment tool [24] and the Methodological Index for Nonrandomized Studies (MINORS) (25) where appropriate. The Cochrane quality assessment tool [24] is developed to assess quality of randomized studies regarding the following domains: selection bias, performance bias, detection bias, attrition bias, and reporting bias. Each domain is rated as low risk, high risk, or unclear risk of bias. The MINORS tool [25] was developed for assessing quality of nonrandomized studies, both comparative and noncomparative. It consists of 12 items concerning study design and methods. Each item is scored as “0” (when the information is not reported), “1” (when the information is reported but it appears inadequate), or “2” (when the information is reported and appears adequate). Items 1–8 refer to all nonrandomized studies, while the remaining 4 items (items 9–12) only apply to comparative studies.

## 3. Results

### 3.1. Search Process

**Figure 1:**
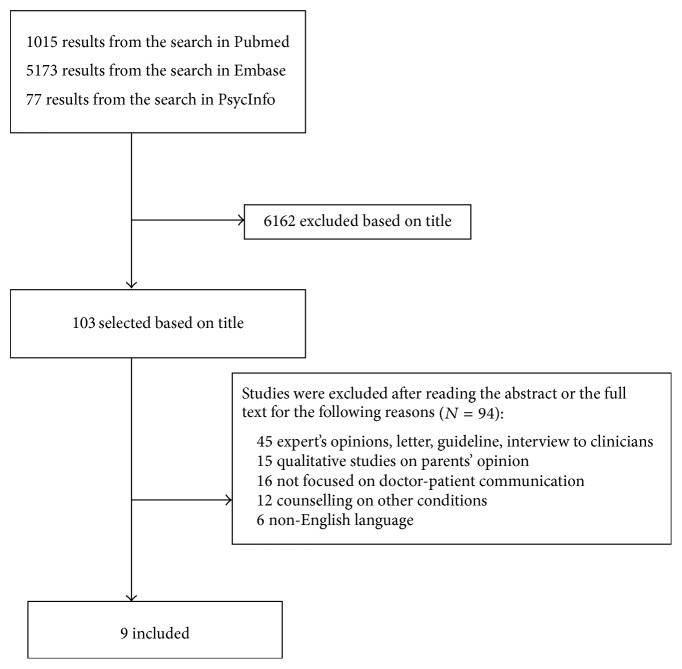
Flow chart of the selection process.

As reported in Figure 1, of the 6,262 papers generated by the preliminary search strategy, 6,162 were excluded by title as they were irrelevant to the study criteria. Abstract and full text were obtained for the remaining 103 papers, of which 45 were excluded because they were expert opinion papers or medical guidelines or letters or interviews to clinicians, 16 were not focused on communication, 15 were qualitative studies on parents' opinions about communication with medical staff, 12 were focused on clinical conditions other than preterm birth, and 6 were excluded because they were not in English language. Finally, a total of 9 studies were included in the review [26–34].

### 3.2. Methodological Quality of the Included Studies

Among the studies included in the review, there were five randomized studies [28, 30–32, 34] and four nonrandomized studies [26, 27, 29, 33]. As reported in Table 2, two randomized studies [30, 31] did not reveal significant risks of bias based on the Cochrane quality assessment tool [24]. Conversely, the other two trials [32, 34] showed a high risk of sampling bias: in one study participants were selected based on voluntary participation and represented a small proportion of the eligible women; in the other study the sample was relatively small. Another randomized study [28] showed risks of bias as the methods of randomization and allocation of participants were not clearly described (risk of selection bias); moreover the evaluator of outcome was not blind to the allocation of participants (detection bias).

Quality assessment of nonrandomized studies based on the MINORS tool [25] is also reported in Table 2. More in detail, all the nonrandomized studies [26, 27, 29, 33] clearly described the aim (item 1). Only one study considered a consecutive sample of participants, while the others focused on a convenience sample or did not report the information (item 2). Two studies recruited a non-real world sample of patients [29, 33]. All the studies followed a protocol established before the beginning of the study (item 3) and included appropriate endpoints (item 4). The assessment of the endpoints was based on interviews to parents or self-reports; thus it could not be blind (item 5). The evaluation of the endpoints was conducted within 24 hours after consultation in two studies [26, 29], and one study included follow-up at three and six months after the consultation (item 6). In one study [27] the proportion of subjects lost at follow-up was considerable (item 7). Only one study [29] reported a sample size calculation (item 8). All the domains concerning the comparative studies [29, 33] were rated as adequate (items 9 through 12).

### 3.3. Characteristics of the Included Studies

As reported in Table 3, all the studies were conducted in the United States [26–34], and one of them was performed in both USA and Netherlands [33]. All the studies were conducted in hospital settings of care, except for one study which recruited participants online and engaged them in a simulated counselling [31]. Notably, three studies can be defined as “*real world*” or “in vivo”, as they considered couples of parents who were really coping with an imminent premature delivery [26, 27, 30, 34]. Conversely, the remaining studies were based on a simulation of counselling [28, 29, 31–33]. More in detail, some studies enrolled a convenience samples of parents with past experience of a child born prematurely [29]; in other studies women with a normal pregnancy [28] or participants in parenting age [31] were asked to imagine they were at risk of imminent delivery. Finally, in other studies obstetricians and neonatologists were asked to counsel a simulated patient at risk of preterm delivery [32, 33].

### 3.4. Procedures of Counselling

#### 3.4.1. Professionals Involved

Four studies reported on which healthcare professionals conducted the counselling: the neonatologist alone [28, 33, 34] or obstetricians and neonatologists in separated sessions [26, 32]. However, none of the studies reported whether the professionals were trained about communication skills or gave information about the amount of previous experience with counselling for preterm delivery.

#### 3.4.2. Tools during Consultation

In four studies the clinicians accompanied oral conversation with visual aids [28, 29], written medical care guidelines [27], gestational-age specific handouts [30], or a pamphlet [34]. Moreover, in some studies the content of conversations was defined based on medical guidelines [30] or local outcome data [28, 31], while all the other studies did not report that the content was set before the consultation.

#### 3.4.3. Timing and Sessions of Consultation

None of the studies specified the length of consultation or the number of sessions, with the exception of two studies applying simulated consultation where each consult was limited to 30 minutes to eliminate time as a variable [32, 33].

#### 3.4.4. Contents of the Consultation

In all the studies, the counselling addressed the clinical issues in terms of description of the risks related to a premature delivery and medical care interventions [26–34]. In addition, some studies focused also on parents' choices about treatment: intrapartum intervention plan [26], resuscitation versus comfort care [26, 28, 31, 33], and neonatal intensive care unit treatments [30, 33].

#### 3.4.5. Style of Communication

One study defined the counselling as “nondirective” [28] and another referred to a consultation “including individualized discussion of parents' values” [27]. Another two studies explicitly referred to the patient-centered model of communication and provided an assessment of centeredness of consultation [32, 33].

### 3.5. Outcomes of the Consultation Practices

#### 3.5.1. Outcome Measures

The recollection of information [26, 28–30, 34], anxiety [30], and satisfaction expressed by parents [27, 34] were the most frequent outcome measures to test effectiveness of counselling. Other studies considered the choices of parents about medical care options [28, 32]. One study was specifically focused on the style of communication in terms of interpersonal skills expressed by physicians in agreement with the shared decision-making model [32].

#### 3.5.2. Main Results of the Studies

The quality of recall was negatively influenced by the level of anxiety [26]. Mothers who received printed information before or during the consultation showed better recall than mothers who received an oral conversation only [28, 30]; moreover they showed a lower level of anxiety after counselling [30]. Conversely, no differences were found when the printed information was provided after the consultation [34]. However, women expressed satisfaction about written medical care guidelines received after counselling [27].

Choices of parents were found to be associated with religion and the overall conception about quality of life [31], while no association was found with the level of details provided by clinicians during counselling, nor with the form under which the information was provided (i.e., with or without visual aids), or with the order in which the treatment options were described [28, 31].

With regard to the style of communication, it was found that information about diagnosis and prognosis was heavily emphasized, while attempts to elicit goals and values were often lacking [32]. Moreover, the influence of cultural factors came to light: physicians of different cultures exhibited different approaches to counselling [33]; at the same time physicians seemed to adapt counselling to the parents' sociocultural variables (i.e., race and insurance status) [32].

## 4. Discussion

The present review showed a research gap in the area of counselling for preterm delivery risk. A small number of studies were included. We found a total of 45 papers reporting experts' opinion and 15 reporting qualitative studies. By contrast, only 9 papers reported quantitative studies. Certainly, qualitative studies provide valuable information to understand the experience of parents and/or healthcare professionals. However, quantitative studies offer the opportunity to provide details about the procedure of counselling. In this sense, this kind of studies is superior as they would represent a stimulus for medical staff interested in improving their procedure of counselling.

Despite the heterogeneity in methods of quantitative studies included in the present review, consistent findings allow identifying two main, although preliminary, clinical implications. First of all, providing written information* before* or* during* the counselling should be considered as a strategy, whenever possible, to improve recollection of information and decrease anxiety [28, 30]. A possible explanation lies in the possibility that, having already read technical info, parents could strengthen the formulation of questions to physicians, and consequently they would obtain more benefit from the consultation. This interpretation seemed to be confirmed by the fact that when the written info was provided* after* the consultation, no improvement was detected in recall, or in anxiety [34]. Moreover, our speculation is in line with several studies conducted in oncology showing that interventions based on providing written info before the consultation resulted effectively in improving the efficacy of counselling [35].

The second implication that can be drawn from the present review refers to the role of parents' beliefs and the relevance of an accurate analysis of this dimension. Indeed, studies showed that, despite the beneficial effect of making information clearer and more accessible, this seemed to have no effect on the parents' choices [28, 31]. These findings are in line with findings of qualitative studies showing that spirituality is strictly related to the decision-making process [2, 36]. However, it should be noted that although parents had stable preferences unaffected by the level of detail provided, sometimes they also showed deep misconceptions [31]. Therefore, understanding parents' preexisting preferences and related beliefs and values is the prerequisite to dispelling misconceptions and, overall, to helping parents in making decisions consistent with their values [31]. This is a central component of PC model (7) and also for SDM [37]. Indeed, the core elements of the patient-centered model refers to communication skills aimed at understanding the patient's perspective [7, 12].

The present review also aimed to verify whether counselling practices were in line with the patient-centered model. A patient-centered consultation implies the following elements: to explore contextual factors (e.g., family, culture, gender, age, socioeconomic status, and spirituality); to explore beliefs, concerns, and expectations about health and illness; to acknowledge and respond to the patient's ideas, feelings, and values [7, 12]. Of nine studies analyzed, two referred in some way to the patient-centered model (6, 27), but only two studies provided an assessment of the centeredness of the consultation [32, 33]. It was found that physicians infrequently explore parents' goal and values [32]. Possible explanations lie in the fear of influencing the parents' choices or in the lack of interpersonal skills [2–4, 38–40]. Moreover, it has been argued that communication skills often receive less attention and emphasis during training than they deserve [14]. Recently, proper education for professionals of perinatal area had been advocated [40].

A third aim of the review was to analyze the methodological quality of the studies, which can be considered from satisfactory to good according to the standardized assessments. However, relevant information about counselling practices was often omitted. One of the main shortcomings was the lack of information about clinicians involved in counselling (e.g., profession, past training on counselling, and level of experience in counselling). This aspect needs further studies considering that obstetricians and neonatologists have different knowledge about premature birth, and this has strong clinical implication in terms of counselling for parents [20, 21]. Moreover, it is interesting to note that often studies included only the mothers rather than both parents. The choice to focus only on mothers could be due to methodological reasons; however it should be considered that organizational aspects and/or the rapid course of maternal disease could hamper the involvement of the father.

When considering the results of the present review, it is worth noting that half of the studies were based on simulated counselling [28, 29, 31–33], rather than being conducted in real world settings of care with parents really facing preterm birth risk. A possible reason explaining the lack of studies is certainly related to the rapid onset of the most frequent conditions predisposing to preterm delivery. Moreover, the emotional status of the parents in such circumstances makes it difficult to introduce to them any study in order to obtain their consent to participate. As for the use of the patient-centered model, it should be also considered that clinicians expressed concerns when this model has to be translated into daily clinical practice; indeed, they pointed out technical and relational difficulties, insufficient training, and fear of losing time [14].

Studies based on simulation, as well studies on parents not really coping with the stressful situation of preterm delivery, can be a valid strategy in order to improve our knowledge in an area that until now has received scant attention. When the physicians of a simulated counselling answered the question “Did the patient encounter feel real?,” the authors concluded that the simulation was highly realistic [32]. Moreover, two studies of the present review were aimed at developing tools to be applied during counselling for preterm delivery; therefore it seems reasonable to preliminary test them on parents with a past experience of preterm delivery or on women with a regular pregnancy rather than on parents really coping with preterm birth [28, 29]. For a similar reason, the use of simulation can be considered appropriated when the primary aim was to compare the contents and styles of counselling delivered by subjects from two cultural backgrounds in a highly standardized scenario [33] or analyze the effects of the level of detail in information and the order of presentation on the treatment choice [31]. Certainly, more research in real world situations is needed to validate these findings.

Finally, it has to be also considered that a total of nine studies were identified, and all the studies were conducted in the USA. The counselling process can be affected by many cultural issues, related to the identification of the decision maker, the role of the communicator, and his attitude towards counselling (issues nicely reviewed in [41]). However, understanding the patient's perspective (by exploring contextual factors, beliefs, concerns, and expectations about health and illness) is a key task in the patient-centered model [12]. At the same time, while in most cases the clinician conducting the counselling is expected to use a nondirective approach, giving a clear recommendation or even persuasion could be ethically justifiable in some cases [41].

In conclusion, despite growing interest for counselling in high-risk pregnancy, the present review suggests that much remains to be done in order to improve this critical process. A progressive advance in the research about this topic is visible; indeed, studies moved from the analysis of technical aspects to encompass also style of communication and interpersonal skills. However, some topics have been completely neglected until now; thus further studies are needed. First of all, there are no studies aimed at analyzing the impact of the use of specific interpersonal skills. Secondly, the recollection of information and level of anxiety were the most frequent outcomes used to test the effectiveness of counselling. By contrast, other crucial aspects receive scant attention. More in detail, from the point of view of parents it would be interesting to analyze also the feeling of trust in medical staff. From the point of view of professionals, it would be interesting to analyze the anxiety and the comfort-discomfort in counselling. Moreover, outcome can be measured also at service level (e.g., in terms of time spent in conversation). Finally, future studies should overcome the dyadic vision of communication by considering the influence of the whole healthcare team on the counselling. Although communication is often conceived in a dyadic vision, parents interact with an entire team rather than refer to one clinician. In this sense, the consensus among colleagues is relevant to guarantee coherence in the information provided to parents in uncertain circumstances such as preterm delivery [42].

## 5. Conclusion

The present review identified a total of nine quantitative studies on counselling about preterm delivery risk. Available findings support (1) providing written information* before* or* during* the consultation as a strategy to improve information achievement by parents and (2) exploring parents' preexisting preferences, beliefs, and values as an essential step to realize the decision-making process. Certainly, more research is needed to validate these findings: the number of studies is small and half of them were based on simulation, rather than a real world setting of care. Future studies should measure the centeredness of conversations and consider the characteristics of clinicians involved in counselling. Finally, cross-cultural studies are needed, considering the influence of cultural variables on the approach to counselling.

## Figures and Tables

**Table 1:** Search strategy.

Database	Search	Resulted records
MEDLINE	“Counseling” Mesh AND (Pregnancy Complications Major Topic OR High-Risk Pregnancy Mesh OR Fetal Diseases Mesh OR Prenatal Care Mesh OR Prenatal Diagnosis Mesh)	*N* = 1015

Embase	Search #1 “pregnacy complication”/exp OR “pregnacy complication” AND (“counselling”/exp OR counselling)	*N* = 1916
	Search #2 “high risk pregnancy”/exp OR “high risk pregnancy” AND (“counselling”/exp OR counselling)	*N* = 539
	Search #3 “fetal disease”/exp OR “fetal disease” AND (“counselling”/exp OR counselling)	*N* = 507
	Search #4 “prenatal diagnosis”/exp OR “prenatal diagnosis” AND (“counselling”/exp OR counselling)	*N* = 5575
	Search #5 “prenatal care”/exp OR “prenatal care” AND (“counselling”/exp OR counselling)	*N* = 2175
	Search #6 #1 OR #2 OR # #3 OR #4 OR #5	*N* = 9323
	Search #7 #6 AND “article”/it	*N* = 5173

PsycInfo	Counselling AND prenatal care	*N* = 31
	Counselling AND prenatal diagnosis	*N* = 37
	Counselling AND pregnancy complications	*N* = 6
	Counselling AND high-risk pregnancy	*N* = 3
	Counselling AND fetal diseases	*N* = 0

**Table 2:** Quality assessment of the included studies.

	Cochrane Quality assessment tool											
	Random sequence generation (selection bias)		Allocation concealment (selection bias)	Blinding of participants and personnel (performance bias)		Blinding of outcome assessment (detection bias)		Incomplete outcome data addressed (attrition bias)		Selective reporting (reporting bias)	Other sources of bias	
Kakkilaya et al., 2011	High risk		High risk	Low risk		High risk		Low risk		Low risk	Low risk	
Muthusamy et al., 2012	Low risk		Low risk	Low risk		Low risk		Low risk		Low risk	Low risk	
Haward et al., 2012	Low risk		Low risk	Low risk		Low risk		Low risk		Low risk	Low risk	
Tucker Edmonds et al., 2014	Low risk		Low risk	Low risk		Low risk		Low risk		Low risk	High risk	
Kett et al., 2016	Low risk		Low risk	Low risk		Low risk		Low risk		Low risk	High risk	

	MINORS tool^*∗*^											
	Item 1	Item 2	Item 3	Item 4	Item 5	Item 6	Item 7	Item 8	Item 9	Item 10	Item 11	Item 12

Zupancic et al., 2002	2	1	2	2	1	2	2	0				
Kaempf et al., 2009	2	2	2	2	1	2	1	0				
Guillen et al., 2012	2	0	2	2	1	2	2	2	2	2	2	2
Geurtzen et al., 2014	2	1	2	2	2	2	2	0	2	2	2	2

^*∗*^The MINORS (25) includes 12 items: clearly stated aim (item 1); inclusion of consecutive patients (item 2); prospective data collection (item 3); endpoints appropriate to the aim (item 4); unbiased assessment of the endpoints (item 5); adequate length of follow-up (item 6); loss to follow-up less than 5% (item 7); calculation of the study size (item 8); adequate control group (item 9); contemporary groups (item 10); baseline equivalence of groups (item 11); adequate statistical analysis (item 12). Items 1–8 refer to all of nonrandomized studies, while additional 4 items only apply to comparative studies. Each item is scored 0 (not reported), 1 (reported but inadequate), or 2 (reported and adequate).

**Table 3:** Characteristics of the included studies.

Author/date/ country	Muthusamy et al. (2012) Texas, USA	Guillen et al. (2012) Pennsylvania, USA	Kakkilaya et al. (2011) Texas, USA

Aims	To assess the effect of providing written information during counselling	To assess outcome of a decision-aid to counsel parents facing premature delivery	To assess outcome of a visual aid to counsel parents facing premature delivery

Study design	Randomized	Nonrandomized	Randomized

Setting	Two hospitals with delivery units and level 3 neonatal intensive care unit	Three urban tertiary care hospitals	A university obstetric clinic serving primarily low-income patients

Patients/participants	Women at risk of preterm delivery (23–34 weeks) randomized as follows: *N* = 30 verbal and written versus* N* = 30 verbal	*N* = 24 couples of parents (13 with past experience with preterm baby and 11 naive)	*N* = 89 women after 28 weeks of a regular pregnancy (44 visual aid; 45 control)

Professional	Not specified	31 clinicians	Neonatologist

Tools applied in counselling	Handout of 5 to 7 pages reporting gestational-age specific information	Visual aids including 6 cards (13 cm × 23 cm) with scripts	Visual aids including graphics, pictures, and short messages

Timing and sessions of counselling	At imminent preterm labour	A simulated counselling session at imminent preterm labour	A simulated counselling session at imminent preterm labour

Style of communication	No description	No description	The counselling was defined as “nondirective”

Contents of counselling	Parental rights to refuse NICU treatment; delivery room care and resuscitation; common treatments and complications; incidence rates of select problems	Size and appearance of preterm baby Survival rates Short term risks	Survival, disability Duration NICU stay Short- and long-term problems Comfort care versus intensive care

Study outcome	(i) Recall of information measured by an ad hoc questionnaire (ii) Anxiety measured by State-Trait (iii) Anxiety inventory (Spielberger 1970)	(i) Knowledge measured by an ad hoc questionnaire administered before and after counselling	(i) Recall of information measured by open-ended oral questions (ii) Parental choices assessed before and after counselling

Main study results	Written information improved knowledge of long-term problems and numerical outcome data, and it also decreased anxiety	Participants found the cards useful and easy to understand. The level of knowledge improved after counselling both for “experienced” parents and “naïve” parents	Women counselled with visual aid recalled more short-term problems, more long-term disability, and longer NICU stay than controls. Attitudes toward resuscitation did not change after counselling in either group

Author/date/ country	Kaempf et al. (2009) Oregon, USA	Zupancic et al. (2002) Ontario, USA	Haward et al. (2012) USA

Aims	To assess the outcome of consensus medical staff guidelines for counselling women at risk of premature birth	To assess outcome of counselling in a routine setting of care	To examine whether choices between comfort care (CC) and intensive care (IC) are affected by the details and the order of presentation

Study design	Nonrandomized	Nonrandomized	Randomized

Setting	Level III for high-risk obstetric and neonatal intensive care unit	Tertiary level referral unit	Online

Patients/participants	*N* = 50 women admitted for potential premature delivery (22–26 weeks)	*N* = 40 women and their partners with diagnosis of preterm labour (23–30 weeks)	*N* = 309 participants in parenting age (18–55 years)

Professional	Not specified	Obstetrician or house staff, and neonatologist separately	The consultation was based on a simulation, particularly on written info (i.e. 2 written pages)

Tools applied in counselling	A consensus about periviability guidelines	Any	The consultation was based on a simulation, particularly on written info (i.e., 2 written pages)

Timing and sessions of counselling	Imminent premature delivery	At admission for preterm delivery	The consultation was based on a simulation, particularly on written info (i.e., 2 written pages)

Style of communication	Family were encouraged to engaged in decision process. The discussion encompassed preferences and values	The format of consultation was left to the discretion of the clinician	The consultation was based on a simulation, particularly on written info (i.e., 2 written pages)

Contents of counselling	(i) Outcome data of premature delivery (ii) Medical care options	(i) Pregnancy complications (ii) Maternal and infant prognosis (iii) Management options	(i) Outcome data of premature delivery (ii) Treatment options (CC or IC)

Study outcome	(i) Satisfaction measured by an ad hoc questionnaire filled 3 days, 6 months, and 18 months after counselling	(i) The level of concordance between parental and clinician about discussed information measured by an ad hoc questionnaire	(i) Choice among CC or IC

Main study results	The women felt comfortable asking questions. About 60% of the mothers mentioned the written guidelines as the most useful information given to them	The agreement score correlated negatively with the level of anxiety. The agreement for obstetric variables was good, while concordance on potential neonatal problems was generally poor	(ii) Order had no effect on final choice (ii) Participants were significantly less likely to choose CC if they were highly religious or valued preservation of life over quality of life

Author/date/ country	Tucker Edmonds et al. (2014) Pennsylvania and Indiana, USA	Geurtzen et al. (2014) California and Netherlands	Kett et al. (2016) Washington, USA

Aims	To assess the feasibility of simulation to test the effect of maternal race and insurance status on shared decision-making (SDM) in periviable counseling	To compare the contents and styles of counseling as delivered by subjects from two cultural backgrounds in a highly standardized scenario	To assess whether a written information provided after the prenatal consultation could improve recall and satisfaction

Study design	Randomized	Nonrandomized	Randomized

Setting	Hospital	Level III neonatal intensive care units	Level III neonatal intensive care unit

Patients/participants	Simulated patients diagnosed with ruptured membranes at 23 weeks	Simulated patient carrying an extremely premature (24 + 6 weeks) fetus	Women at risk of preterm delivery (22–30 weeks) randomized as follows: *N* = 18 verbal and written versus* N* = 18 verbal

Professional	*N* = 37 obstetricians *N* = 15 neonatologists	*N* = 22 neonatologists (11 American and 11 Dutch)	Neonatologists

Tools applied in counselling	Any	Any	7-page pamphlet reporting: definition of preterm birth, causes, what to expect in the delivery room, and health problems encountered by preterm infants

Timing and sessions of counselling	Each consult was limited to 30 minutes to eliminate time as a variable	Each consult was limited to 30 minutes to eliminate time as a variable	At imminent preterm labour

Contents of counselling	(i) Outcome data of premature delivery (ii) Medical care options (iii) Parents' goals and values	(i) Survival rates (ii) Physiologic morbidities (iii) Options for care: comfort care versus treatment (i.e., partial or maximal life support)	Not described

Style of communication	The level of shared decision-making measured by Braddock scale coding applied to verbatim audio registrations	The consultations were video-recorded and the interpersonal skills were scored using a standardized instrument of coding	No description

Study outcome	The level of SDM	The content and the style of counseling	(i) Recall of the factual information (ii) Satisfaction with the consultation

Main study results	(i) Information regarding diagnosis and prognosis was heavily emphasized, while attempts to elicit goals and values were often lacking (ii) SDM occurs differentially based on patients' race and insurer	(i) American and Dutch neonatologists diverged in the discussed and emphasized options for immediate care in the delivery room	The two groups did not differ in factual recall (within 72 h) or satisfaction with the prenatal consultation
